# Analysis of adaptive responses of *Pinus pinaster* to changing environmental conditions in the Mediterranean region

**DOI:** 10.1186/1753-6561-5-S7-P87

**Published:** 2011-09-13

**Authors:** Marina de Miguel, Angeles Guevara, Nuria de María, Enrique Sáez, Luis-Manuel Díaz, Carmen Collada, Alvaro Soto, Pedro Perdiguero, José-Antonio Cabezas, David Sánchez-Gómez, Ismael Aranda, Teresa Cervera

**Affiliations:** 1 Dpto. Ecología y Genética Forestal, CIFOR-INIA, Ctra. de La Coruña km 7, 28040 Madrid, Spain; 2 Dpto. de Biotecnología, ETSIM (UPM), Ciudad Universitaria s/n, 28040 Madrid, Spain; 3 Dept. Silvopasticultura, ETSIM (UPM), Ciudad Universitaria s/n, 28040 Madrid, Spain; 4 Dept. de Silvopasticultura, ETSIM (UPM), Ciudad Universitaria s/n, 28040 Madrid, Spain; 5 Dpto. Investigación Agroalimentaria, IMIDRA, Finca El Encín. Ctra A-2, Km38.200, 28800 Alcalá de Henares, Spain; 6 Dpto. Silvopasticultura, ETSIM (UPM), Ciudad Universitaria s/n, 28040 Madrid, Spain

## 

Recent climate evolution studies highlight the progressive temperature increase and prevalence of seasonal drought, with specially incidence in the Mediterranean region. Although conifers are very important species regarding forest conservation, sustainability and productivity, given the large forest surface they cover in Spain and their active role in preventing soil erosion and desertification, we know little about the molecular mechanisms which control adaptation in this ancient taxonomic group.

The work is focused on the study of adaptive responses of maritime pine (*Pinus pinaster* Ait.), one of the most important gymnosperm species in the Mediterranean region. To better understand these processes, we designed a strategy which integrates several approaches:

- Phenotypic characterization in response to drought, evaluating functional (physiological) and morphological parameters in a full-sib family originated from a controlled cross between two progenitors showing an *a priori* contrasting response to drought. In order to improve the phenotypic estimates, clonal material was generated by vegetative propagation and several ramets per clone were analyzed per experiment. Two experiments have been already developed: a) Plants from a complete full-sib family were exposed to severe drought for a short time; b) Selected clones showing contrasting behavior in their response to drought were subjected to a moderate drought for long time. The latest experiment was designed to better understand the functional mechanisms of drought tolerance. In 2011 a third experiment will be developed to evaluate survival capacity.

- Molecular characterization of the adaptive response, including:

1. Transcriptome analysis. ESTs collections associated with different tissues and growth conditions. We developed: a) Subtractive cDNA libraries to study the response of clonal material subjected to controlled hydric stress. A total of 386 unigenes have been identified from the SSH library (Sanger sequencing), 351 of them presumably corresponding to nuclear sequences. We have selected a set of 67 reliable candidate genes significantly upregulated by PEG-induced water stress, according to a microarray analysis: 45, 29 and 29 from roots, stems and needles, respectively. Functional classification of the unigenes, based on the BLAST homology analysis, showed that the largest group corresponds to genes involved in metabolism (16%). Up to 42% of these are related with carbohydrate metabolism, consistently with the role played by sugar accumulation in drought tolerance. b) cDNA library made of mRNAs extracted from buds collected from January to April, to dissect bud burst in this species. A total of 9,000 ESTs were sequenced by Sanger (CT574594 - CT583294) in collaboration with C. Plomion, UMR BIOGECO. c) A cDNA library made of mRNAs from multiple tissues (needles, stems and roots) and growing conditions (including drought stress and hormone treatments – auxin and cytokinin) collected from full-sib progeny individuals (collaboration with C. Díaz-Sala, UAH). The cDNA library was constructed by EVROGEN and used as template for Roche GS-FLX Titanium high throughput sequencing (Lifesequencing). A total of 22,427 isotigs/contigs were assembled from 1,218,000 reads. 15,722 isotigs/contigs were annotated and a total of 9,085 proteins detected.

2. Characterization of full-length cDNAs (FLcDNA). Complete and accurate annotation of functional genes rely largely on the information of FL-cDNAs since eukaryotic genes often have differential splicing of introns and unpredictable transcription starting sites, making gene prediction less accurate. In order to characterize FLcDNAs, a pool of approximately 5.000 PCR amplified inserts from selected cDNA clones (based on cDNA length versus annotation) from different EST libraries was generated. This pool of inserts was sequenced using Roche GS-FLX Titanium. A total of 3,259 isotigs from 573,242 aligned reads were obtained. This work was carried out in collaboration with S. Fluch (ARC) and C. Plomion (UMR BIOGECO).

3. SNP identification and analysis. Two sources of SNPs were used: 1) SNPs detected in a set of re-sequenced candidate genes (*in vitro* SNPs) used to design a 384 and a 1536 Golden Gate arrays for diversity analysis (to be used in association studies) and construction of genetic maps, and 2) SNPs detected from the third cDNA library described in the transcriptome analysis section, to design a 1536 Golden Gate array for construction of genetic maps as well as a 7600 Infinium assay for diversity and mapping purpose (the latest in collaboration with C. Plomion).

4. Construction of genetic linkage maps using dominant (SAMPLs), as well as co-dominant (microsatellites, ESTPs and SNPs) markers. Two INIA and two INRA mapping progenies will be used to construct species genetic map of *Pinus pinaster*. For this purpose, a set of common markers including SSRs, ESTPs and SNPs from three Illumina assays (384 Vera Code; 1536 Golden Gate; 7600 Infinium assays) will be performed to genotype 100 individuals per mapping progeny. Comparative mapping with other conifers will also be addressed.

5. Association studies. Association analyses are in progress by looking for association between phenotypes (growth, drought stress response) and genotypes (SNP genotypes) of 22 populations (each represented by 20 to 30 trees), spanning the natural distribution of the species (collaboration with C Plomion, UMR BIOGECO) and a metapopulation from Central Spain which consists of 5 populations (each represented by 20 to 30 trees; collaboration with C. Plomion, UMR BIOGECO and Reiner Finkeldey, UGO). QTL analysis is carried out to identify genome regions involved in the genetic control of maritime pine response to hydric stress, growth and biomass production based on association studies between genotypes of mapping populations and their corresponding phenotypes.

6. Maritime pine genome sequence. De novo sequencing of *Pinus pinaster* genome using haploid DNA will be carried out using current next-generation DNA sequencing technologies and a shotgun strategy. This approach includes: 1) >30x genome coverage based on paired-end (short-insert libraries) and additional >10x genome coverage base on mate-pairs (long-insert libraries) using HiSeq2000 sequencing; 2) use of mate-pairsequenced with GS FLX Titanium to construct longer scaffolds; 3) use of transcriptomics data described in sections “a” including FLcDNAs (section “2”) to confirm results; and 4) genome assembly and annotation. This research results from the collaborative work between Spanish groups from UMA, PAB, UV, UPM, CNAG, BSC, Lifesequencing and CIB-CSIC coordinated by CIFOR-INIA and UAH and it is supported by the Spanish Ministry of Science and Innovation.

